# Supplementary material for: Exploring the molecular structures that confer ligand selectivity for galanin type II and III receptors
Source: PLoS One. 2020 Mar 31;15(3):e0230872. doi: 10.1371/journal.pone.0230872 (PMC7108740; doi:10.1371/journal.pone.0230872)
Supplement: S1 Text — (DOCX) [file pone.0230872.s001.docx]

**S1 Text. Supplementary materials and methods**

**HiBiT assay**

Membrane expression of receptors was measured by using Nano-Glo HiBiT (Hibit sequence; ATGGTGAGCGGCTGGCGGCTGTTCAAGAAGATTAGC) extracellular detection system acquired from Promega Corporation (Cat No. N2421). HEK293T cells were seeded in 96-well plates as a density of 2.0 x 10^4^ cells per well. Prepared mixture with 0.5 ng if SmBiT-high affinity-receptor construct (WT; GALR2, GALR3, chimeric receptors; GALR2/3a, GALR2/3b, GALR2/3c, GALR2/3d, GALR2/3e, GALR2/3f, GALR3/2a, GALR3/2b, GALR3/2c, GALR3/2d, GALR3/2e, GALR3/2f) and 0.2 μl of Lipofectamine 2000 (Invitrogen) were transfected into each cell according to the manufacturer’s instructions. Approximately 24 h after transfection, 100 μl of Nano-Glo HiBiT extracellular reagents (1 μl of LgBit protein + 2 μl Substrate + 97 μl of Nano-Glo HiBiT buffer) were added and maintained to equilibrate for 4 min at room temperature without mixing. Then luminescence values were recorded immediately (Synergy 2 Multi-Mode Microplate Reader BioTek).

**Molecular Dynamics**

The MD simulations were carried out using Desmond simulation package of Schrödinger LLC (Schrödinger, NY, USA). The *NPT* ensemble with the temperature 300 K and a pressure 1 bar was applied in all runs. The simulation length was 30 ns with a relaxation time 1 ps for each peptide. The OPLS3e force field parameters were used in all simulations. The long-range electrostatic interactions were calculated using the particle mesh Ewald method. The cutoff radius in Coulomb interactions was 9.0 Å. The TIP3P water model was used and 150 mM NaCl was added to the solvent model. The Martyna–Tuckerman–Klein chain coupling scheme using isotropic style with a coupling constant of 2.0 ps was used for the pressure control and the Nosé–Hoover chain coupling scheme for the temperature control. Nonbonded forces were calculated using an r-RESPA integrator where the short-range forces were updated every step and the long-range forces were updated every three steps. The trajectories were saved at 30 ps intervals for analysis. MD trajectory analysis was used for the clustering of peptides.
