# Supplementary figures and images for: Exploring the molecular structures that confer ligand selectivity for galanin type II and III receptors
Source: PLoS One. 2020 Mar 31;15(3):e0230872. doi: 10.1371/journal.pone.0230872 (PMC7108740; doi:10.1371/journal.pone.0230872)

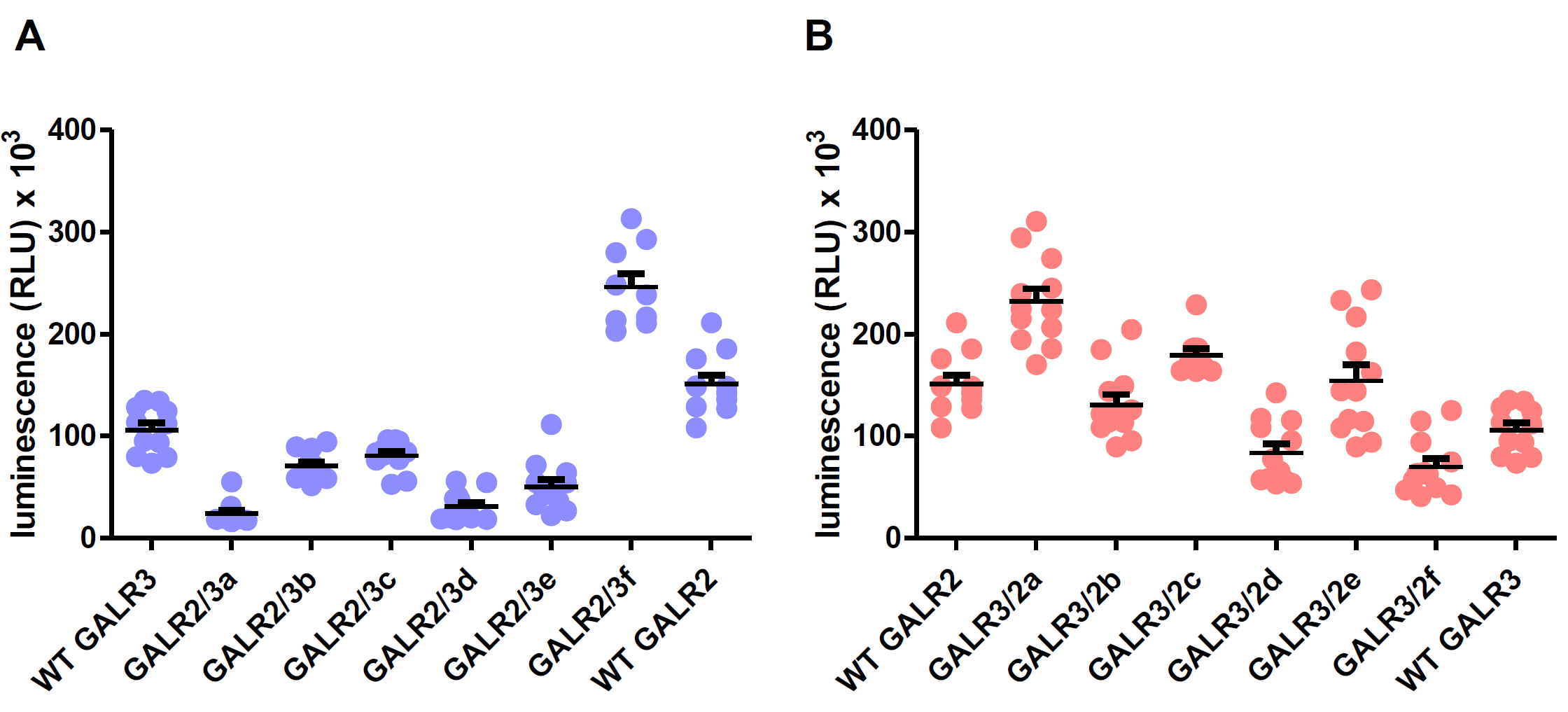

Supplement: S1 Fig — SmBit-tagged chimeric receptors were expressed and treated with LgBiT. Bioluminescence was measured in cells expressing WT GALR2, WT GALR3, chimeric GALR2/3 (A), or chimeric GALR3/2 (B) receptors. Data are presented as the mean ± SE. (TIF) [file pone.0230872.s002.tif]

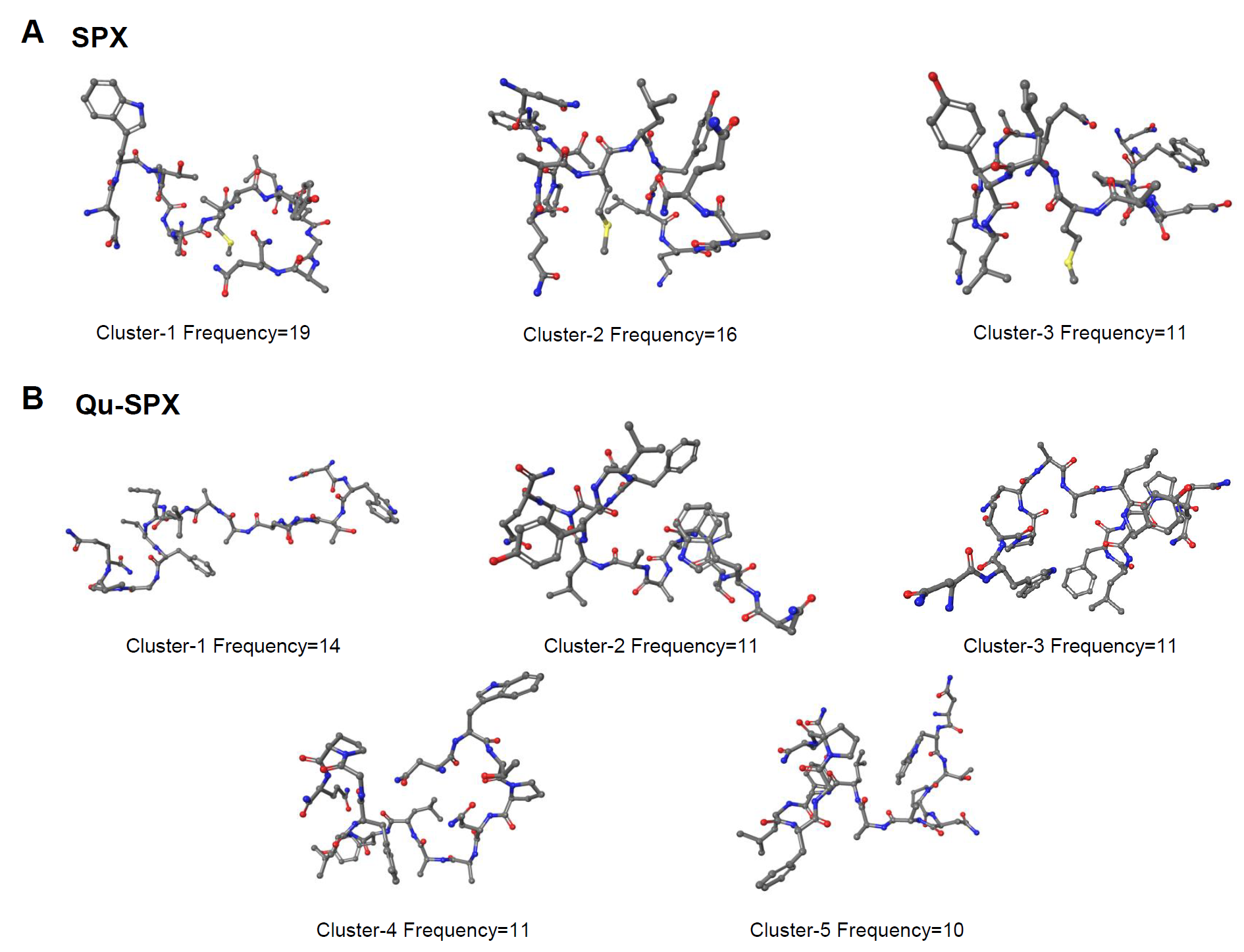

Supplement: S2 Fig — Solution structures of SPX (A) and Qu-SPX (B) are predicted using a molecular dynamics (MD) simulation method. MD trajectory analysis was used for the clustering of peptides. Structures with more than 10 frequencies out of 1000 snapshots are displayed. (TIF) [file pone.0230872.s003.tif]
