# Supplementary material for: Exploring the molecular structures that confer ligand selectivity for galanin type II and III receptors
Source: PLoS One. 2020 Mar 31;15(3):e0230872. doi: 10.1371/journal.pone.0230872 (PMC7108740; doi:10.1371/journal.pone.0230872)
Supplement: S1 Table — (DOCX) [file pone.0230872.s004.docx]

**S1 Table. Differential responses of GALR2/3 and GALR3/2 chimeric receptors to ligands.**

| **Receptor** | **SPX** | **Qu-SPX** | **Receptor** | **SPX** | **Qu-SPX** |
| --- | --- | --- | --- | --- | --- |
|  | **EC_50_ [nM]** | **EC_50_ [nM]** |  | **EC_50_ [nM]** | **EC_50_ [nM]** |
|  | **Emax**  **(fold induction)** | **Emax**  **(fold induction)** |  | **Emax**  **(fold induction)** | **Emax**  **(fold induction)** |
| GALR2 | 45.70±11.03 | 87.09±19.48 | GALR3 | 114.81±17.09 | N.A |
|  | 16.28 | 16.26 |  | 31.99 |  |
| GALR2/3a | 50.11±12.09 ^a^ | N.A | GALR3/2a | 234.42±5.33 ^b^ | 457.08±110.35 ^b^ |
|  | 12.11 |  |  | 44.84 | 52.73 |
| GALR2/3b | 35.48±6.64 ^a^ | N.A | GALR3/2b | 588.84±121.10 ^b^ | 1122.01±188.76 ^b^ |
|  | 37.21 |  |  | 20.84 | 28.06 |
| GALR2/3c | 45.70±8.55 ^a^ | N.A | GALR3/2c | N.A | N.A |
|  | 23.15 |  |  |  |  |
| GALR2/3d | >1000 | N.A | GALR3/2d | 109.64±32.02 ^b^ | 954.99±174.74 ^b^ |
|  | 12.89 |  |  | 21.82 | 12.18 |
| GALR2/3e | 616.59±280.07 ^a^ | N.A | GALR3/2e | 398.10±59.26 ^b^ | N.A |
|  | 3.11 |  |  | 39.68 |  |
| GALR2/3f | N.A | N.A | GALR3/2f | N.A | N.A |

N.A.: Not applicable
