# Supplementary material for: Exploring the molecular structures that confer ligand selectivity for galanin type II and III receptors
Source: PLoS One. 2020 Mar 31;15(3):e0230872. doi: 10.1371/journal.pone.0230872 (PMC7108740; doi:10.1371/journal.pone.0230872)
Supplement: S2 Table — (DOCX) [file pone.0230872.s005.docx]

**S2 Table. Responses of GALR3-like chimeric receptors single amino acid-substituted mutant peptides.**

| **Receptor**  EC_50_ [nM] | **SPX** | **N^5^-SPX**  **(Q→N)** | **A^7^-SPX**  **(M→A)** | **F^11^-SPX**  **(K→F)** | **P^13^-SPX**  **(A→P)** |
| --- | --- | --- | --- | --- | --- |
| **GALR2** | 45.70±11.03 | 87.72±10.62 | 40.73±13.19 | 47.86±11.55 | 33.11±6.19 |
| **GALR3** | 114.81±17.09 | 398.10±96.11 | 467.73±69.62 | 489.77±118.24 | 333.13±55.70 |
| **GALR2/3a** | 48.97±10.07 | 549.54±122.96^a^ | 912.01±266.35^a^ | 616.59±126.81^a^ | 575.43±177.33^a^ |
| **GALR2/3b** | 30.90±5.78 | 309.02±63.55^a^ | 446.68±107.83^a^ | 371.53±62.50^a^ | 758.57±221.54^a^ |
| **GALR2/3c** | 14.79±3.30 | 37.15±10.85^b^ | 97.72±26.92^b^ | 218.77±63.89^a^ | 407.38±105.38^a^ |

The EC_50_ values are presented as mean ± S.E.

a, P<0.05 vs. WT SPX

b, P<0.05 vs. GALR2/3b chimeric receptor
