# Supplementary material for: Exploring the molecular structures that confer ligand selectivity for galanin type II and III receptors
Source: PLoS One. 2020 Mar 31;15(3):e0230872. doi: 10.1371/journal.pone.0230872 (PMC7108740; doi:10.1371/journal.pone.0230872)
Supplement: S3 Table — (DOCX) [file pone.0230872.s006.docx]

**S3 Table. Responses of GALR3 mutant receptors to**

**N^5^-mutant and A^7^-mutant peptides**

| **Chimeric receptors**  EC_50_ [ nM ] | **SPX** | **N^5^-SPX** | **A^7^-SPX** |
| --- | --- | --- | --- |
| GALR3/2_[TM3]_ | 25.11±3.74 | 89.13±16.68 ^a, b^ | 48.98±12.67 ^a,b^ |
| GALR3/2_[TM4]_ | 42.66±6.35 | 309.03±51.99 ^a^ | 338.84±87.66 ^a,b^ |
| **Single mutant receptors**  EC_50_ [ nM ] | **SPX** | **N^5^-SPX** | **A^7^-SPX** |
| L^100^F | 66.07±13.59 | 288.40±84.23 ^a^ | 269.15±45.28 ^a^ |
| Y^103^F | 32.36±10.48 | 676.08±126.54 ^a^ | 302.00±72.91 ^a^ |
| Y^107^H | N.A. | N.A | N.A |
| **Double/Triple mutant receptors**  EC_50_ [ nM ] | **SPX** | **N^5^-SPX** | **A^7^-SPX** |
| L^100^F, Y^103^F | 42.66±9.54 | 173.78±44.96 ^a,b^ | 154.88±45.23 ^a,b^ |
| L^100^F, Y^107^H | 38.90±7.28 | 154.88±40.07 ^a,b^ | 69.18±20.20 ^b^ |
| Y^103^F, Y^107^H | N.A. | N.A. | N.A. |
| L^100^F, Y^103^F, Y^107^H | 30.90±9.03 | 83.18±26.94 ^a,b^ | 64.57±12.09 ^a,b^ |

The EC_50_ values are presented as mean ± S.E.

a, P<0.05 vs. WT SPX

b, P<0.05 vs. WT GALR3

N.A.: Not applicable
