# Supplementary material for: Exploring the molecular structures that confer ligand selectivity for galanin type II and III receptors
Source: PLoS One. 2020 Mar 31;15(3):e0230872. doi: 10.1371/journal.pone.0230872 (PMC7108740; doi:10.1371/journal.pone.0230872)
Supplement: S4 Table — (DOCX) [file pone.0230872.s007.docx]

**S4 Table.** **Responses of GALR3 mutant receptors to F^11^-mutant and P^13^-mutant peptides**

| **Chimeric receptors**  EC_50_ [ nM ] | **SPX** | | **F^11^-SPX** | | **P^13^-SPX** |
| --- | --- | --- | --- | --- | --- |
| GALR3/2_[TM5]_ | 20.89±4.29 | | 354.81±85.66 ^a^ | | 60.26±10.14 ^a,b^ |
| GALR3/2_[TM6]_ | N.A. | | N.A. | | N.A. |
| **Single mutant receptors**  EC_50_ [ nM ] | | **SPX** | | **P^13^-SPX** | |
| L^184^M | | 46.77±15.87 | | 436.52±89.78 ^a^ | |
| V^186^I | | 141.25±45.75 | | 512.86±158.04 ^a, b^ | |
| A^187^C | | 114.82±27.72 | | 549.54±151.43 ^a, b^ | |
| A^190^V | | 77.62±18.74 | | 467.74±104.65 ^a, b^ | |
| A^191^F | | 91.20±30.95 | | 467.74±104.65 ^a^ | |
| G^192^S | | 35.48±12.57 | | 354.81±109.34 ^a^ | |
| **Various mutant receptors**  EC_50_ [ nM ] | | **SPX** | | **P^13^-SPX** | |
| L^184^M,V^186^I,A^187^C | | 114.82±2.61 | | 457.09±118.24 ^a, b^ | |
| L^184^M,V^186^I,A^190^V | | 48.98±16.61 | | 323.59±89.17 ^a^ | |
| A^190^V,A^191^F,G^192^S | | 33.88±10.98 | | 123.03±39.85 ^a, b^ | |
| L^184^M,V^186^I,A^187^C,  A^190^V,A^191^F | | 50.12±16.23 | | 141.25±36.54 ^a, b^ | |
| L^184^M,V^186^I,A^187^C,  A^190^V,G^192^S | | 33.88±9.89 | | 144.54±24.31 ^a, b^ | |
| L^184^M,V^186^I,A^187^C,  A^190^V,A^191^F,G^192^S | | 25.70±7.92 | | 112.20±25.10 ^a, b^ | |

The EC_50_ values are presented as mean ± S.E.

a, P<0.05 vs. WT SPX

b, P<0.05 vs. WT GALR3
