# Supplementary material for: Exploring the molecular structures that confer ligand selectivity for galanin type II and III receptors
Source: PLoS One. 2020 Mar 31;15(3):e0230872. doi: 10.1371/journal.pone.0230872 (PMC7108740; doi:10.1371/journal.pone.0230872)
Supplement: S5 Table — (DOCX) [file pone.0230872.s008.docx]

**S5 Table.** **Responses of GALR3 mutant receptors to F^11^-mutant peptide**

| **Chimeric receptors**  EC_50_ [ nM ] | **SPX** | **F^11^-SPX** |
| --- | --- | --- |
| GALR3/2_[ECL1]_ | 30.90±9.03 | 257.04±70.83 ^a^ |
| GALR3/2_[ECL2]_ | N.A. | N.A. |
| GALR3/2_[ECL3]_ | 39.81±11.62 | 51.29±17.40 ^b^ |
| **Single mutant receptors**  EC_50_ [ nM ] | **SPX** | **F^11^-SPX** |
| A^264^P | 273.87±75.96 | 588.84±199.80 ^a^ |
| F^265^P | >1000 | 457.09±118.24 ^a,^ |
| S^266^T | 38.01±10.48 | 199.53±29.70 ^a, b^ |
| P^267^R | 114.82±48.75 | 562.34±173.30 ^a^ |
| C^272^L | 28.84±6.96 | 66.07±22.41 ^a, b^ |

The EC_50_ values are presented as mean ± S.E.

a, P<0.05 vs. WT SPX

b, P<0.05 vs. WT GALR3

N.A.: Not applicable
