# Supplementary material for: Exploring the molecular structures that confer ligand selectivity for galanin type II and III receptors
Source: PLoS One. 2020 Mar 31;15(3):e0230872. doi: 10.1371/journal.pone.0230872 (PMC7108740; doi:10.1371/journal.pone.0230872)
Supplement: S6 Table — (DOCX) [file pone.0230872.s009.docx]

**S6 Table.** **Responses of GALR3 septuple-mutant receptor** **to Qu-SPX**

| **Luciferase assay**  **EC_50_ [nM]** | **GALR3** | **GALR3 septuple** |
| --- | --- | --- |
| SPX | 114.82±21.49 | 4.07±1.12 |
| Qu-SPX | N.A. | 1096.48±163.22 |
| **cAMP assay**  **EC_50_ [nM]** | **GALR3** | **GALR3 septuple** |
| SPX | 117.49±51.42 | 77.62±38.72 |
| Qu-SPX | N.A. | 794.33±357.81 |

N.A.: Not applicable
