# Supplementary material for: Exploring the molecular structures that confer ligand selectivity for galanin type II and III receptors
Source: PLoS One. 2020 Mar 31;15(3):e0230872. doi: 10.1371/journal.pone.0230872 (PMC7108740; doi:10.1371/journal.pone.0230872)
Supplement: Attachment — Submitted filename: Response to Reviewers.docx [file pone.0230872.s010.docx]

February 29, 2020

Dear Editor

We thank you for allowing us to revise our manuscript entitled “Exploring the molecular structures that confer ligand selectivity for galanin type Ⅱ and Ⅲ receptors” [PONE-D_19_33974]. We carefully revised the manuscript to incorporate comments and concerns raised by the editor and reviewers. The major changes are as follows.

1. To address the comment from the editor and reviewer 2, we conducted molecular dynamics (MD) simulation study to predict the solution structures of SPX and Qu-SPX. MD-resolved structures of SPX and Qu-SPX differ each other, although both peptides may not have stable three-dimensional structures in solution. This result raises a possibility that conformations of Qu-SPX different from those of SPX may hamper full recovery of the potency toward the septuple-mutant GALR3. The result is shown in S2 Fig. As this experiment was conducted by Dr. Chul-Soon Lee we (all authors) decided to include him as a co-author of the article.
2. The GAL sequence is included in Fig 1 as reviewer 1 suggested.
3. The manuscript is revised to meet PLOS ONE's style requirements.

**Response to reviewer #1**

We appreciate your valuable and helpful comments on our manuscript.

Point 1: English needs significant improvement. For example, in the abstract they state "we identified residues in GALR2 that "attract" SG2A and residues in GALR3 that "avoid" SGA2. How can amino acids "attract" or "avoid" ligands? Suggest using scientific terms such as interact, inhibit etc.

→We used scientific terms in the revised manuscript. Please see page 2 line26, and line 31. Please see page 4 line 86 line. Please see page 17 line 465, and 466.

Point 2 : Should show sequence of GAL peptide in Figure 1.

→We added the GAL peptide sequence in the revised Figure 1 (please see Page 5 line 102 and line 104).

Point 3 : How similar is the Qu-SPX peptide compared to GAL? Is SPX different from GAL peptide in only the four amino acids replaced or would more complete substitution with amino acids from GAL peptide in Qu-SPX rescue GALR3 activity for the Qu-SPX peptide? This might explain why the GALR3 septuple mutant shows very subtle activity towards Qu-SPX.

→ We agree on reviewer’s opinion that additional substitutions with amino acids from GAL peptide in Qu-SPX rescue GALR3 activity more. In our previous report (Reyes-Alcaraz et al., 2016, Ref# 26), we explained in detail that four amino acids replacement (Asn^5^, Ala^7^, Leu^11^/Phe^11^, and Pro^13^ substitution) in SPX completely abolished potency to GALR3 while mutations in other residues of SPX did not influence GALR2/GALR3 selectivity. Therefore, we focused on these 4 residue substitutions. We include these points in the revised manuscript. (Please see page 4 line 80).

In addition, we conducted molecular dynamics (MD) simulation analysis to predict the solution structures of SPX and Qu-SPX. The result shows that structures of SPX and Qu-SPX differ each other, although both peptides may not have stable three-dimensional structures in solution. This result raises a possibility that conformations of Qu-SPX different from those of SPX may hamper full recovery of the potency toward the septuple-mutant GALR3. This notion is included in the revised text. Please see **S1 text** for Molecular Dynamics and **S2 Fig**.

**Response to reviewer #2**

We appreciate your valuable and helpful comments on our manuscript.

Point 1: The main flaw of the manuscript regards the interpretation of the results. In the manuscript, the discussion is carried out considering that the observed differences in the binding affinity of the diverse analogs can be interpreted in terms of ligand-receptor interactions. However, peptides are flexible and their conformational profile is affected by the sequence. We may consider that one amino acid substitution may not change much the conformational profile of the analogs, but four as in Qu-SPX is risky. Accordingly, the discussion should be carried out considering a loss of an interaction and/or to a different ligand conformational behavior. These considerations are lacking in the present manuscript

→ To address this point, we tried to predict the solution structures of SPX and Qu-SPX using a molecular dynamics (MD) simulation method. MD trajectory analysis was used for the clustering of peptides. Indeed, MD-resolved structures of SPX and Qu-SPX differ each other, although both peptides may not have stable three-dimensional structures in solution. This result raises a possibility that conformations of Qu-SPX different from those of SPX may hamper full recovery of the potency toward the septuple-mutant GALR3. This notion is included in the revised text. Please see **S1 text** for Molecular Dynamics and **S2 Fig**.

Minor details: “The strong response to WT SPX was retained by all of the GALR2/3 series a to c chimeric receptors, whereas it was drastically weakened in the GALR2/3 series d and e chimeric receptors” requires revision. I suggest referring to SPX in the same way all through the manuscript. For example, WT SPX

→ WT SPX is changed to SPX throughout the revised manuscript.

Thank you again for your editorial effort and time for this manuscript.

Sincerely yours,

Jae Young Seong, Ph.D., Professor

Lab of G protein Coupled Receptors

Korea University College of Medicine

Seoul 136-705

KOREA

Tel: +82-2-2286-1090

Fax: +82-2-921-4355

E-mail: jyseong@korea.ac.kr
